# Evaluation of Response Processes to the Danish Version of the Dutch Multifactor Fatigue Scale in Stroke Using the Three-Step Test-Interview

**DOI:** 10.3389/fnhum.2021.642680

**Published:** 2021-05-06

**Authors:** Frederik L. Dornonville de la Cour, Anne Norup, Trine Schow, Tonny Elmose Andersen

**Affiliations:** ^1^BOMI Brain Injury Rehabilitation Center, Roskilde, Denmark; ^2^Department of Psychology, Faculty of Health Sciences, University of Southern Denmark, Odense, Denmark; ^3^Neurorehabilitation Research and Knowledge Centre, Rigshospitalet, Copenhagen, Denmark

**Keywords:** post-stroke fatigue, Dutch Multifactor Fatigue Scale, validity, cognitive interviewing, framework analysis, item response processes, think aloud

## Abstract

Validated self-report measures of post-stroke fatigue are lacking. The Dutch Multifactor Fatigue Scale (DMFS) was translated into Danish, and response process evidence of validity was evaluated. DMFS consists of 38 Likert-rated items distributed on five subscales: Impact of fatigue (11 items), Signs and direct consequences of fatigue (9), Mental fatigue (7), Physical fatigue (6), and Coping with fatigue (5). Response processes to DMFS were investigated using a Three-Step Test-Interview (TSTI) protocol, and data were analyzed using Framework Analysis. Response processes were indexed on the following categories: (i) “*congruent*,” response processes were related to the subscale construct; (ii) “*incongruent*,” response processes were not related to the subscale construct; (iii) “*ambiguous*,” response processes were both congruent and incongruent or insufficient to evaluate congruency; and (iv) “*confused*,” participants did not understand the item. Nine adults were recruited consecutively 10–34 months post-stroke (median = 26.5) at an outpatient brain injury rehabilitation center in 2019 [five females, mean age = 55 years (*SD* = 6.3)]. Problematic items were defined as <50% of response processes being congruent with the intended construct. Of the 38 items, five problematic items were identified, including four items of Physical fatigue and one of Mental fatigue. In addition, seven items posed various response difficulties to some participants due to syntactic complexity, vague terms, a presupposition, and a double-barrelled statement. In conclusion, findings elucidate the interpretative processes involved in responding to DMFS post-stroke, strengthen the evidence base of validity, and guide revisions to mitigate potential problems in item performance.

## Introduction

Fatigue is a common complaint following stroke (Christensen et al., 2008; Duncan et al., 2012, 2014; Cumming et al., 2016) and interferes with health-related quality of life (Naess et al., 2006; van de Port et al., 2006), participation in daily activities (Röding et al., 2003; Naess et al., 2005; White et al., 2012; Maaijwee et al., 2015), and return to work (Lock et al., 2005; Andersen et al., 2012). The experience of fatigue is inherently subjective (Aaronson et al., 1999; Staub and Bogousslavsky, 2001a), and stroke survivors’ perspectives reveal that fatigue is a heterogeneous condition with several characteristics (Eilertsen et al., 2013). Fatigue is a nonspecific symptom with multiple potential causes and contributing factors (Krupp, 2003), and it is closely related to other common post-stroke conditions such as depression (Ponchel et al., 2015; Douven et al., 2017; Dornonville de la Cour et al., 2020) and sleep disorders (Aarnes et al., 2020). Several definitions of fatigue have been proposed (both generic and disease-specific) (Aaronson et al., 1999; Staub and Bogousslavsky, 2001a) without consensus on a standard definition, and various subdomains of fatigue are in use without clear terminology (Kluger et al., 2013). Examples are objective vs. subjective fatigue (Staub and Bogousslavsky, 2001b), pathological vs. normal fatigue (Aaronson et al., 1999), central vs. peripheral fatigue (Chaudhuri and Behan, 2004), primary vs. secondary fatigue (DeLuca, 2005), and mental fatigue, physical fatigue, psychological fatigue, and somatic fatigue (Staub and Bogousslavsky, 2001b).

The complexity of fatigue and the lack of a standard definition hamper efforts to develop standard assessment tools of self-reported fatigue (Aaronson et al., 1999; Kluger et al., 2013). A large number of self-report scales addressing fatigue are available (Whitehead, 2009). However, most scales were developed for other populations than stroke such as multiple sclerosis or cancer patients (Whitehead, 2009), and psychometric properties of fatigue scales are not well documented in neurological conditions (Tyson and Brown, 2014). Furthermore, fatigue scales used in stroke populations address a wide variety of attributes and lack overlap in item contents, indicating disparity in the aspects of fatigue represented by different fatigue scales (Skogestad et al., 2019).

The Dutch Multifactor Fatigue Scale (DMFS) was developed to assess the nature and impact of long-lasting fatigue (>6 months) following acquired brain injury (ABI) specifically, including the way respondents cope with fatigue (Visser-Keizer et al., 2015). DMFS comprises 38 items distributed on five subscales: Impact of fatigue (11 items), Signs and direct consequences of fatigue (nine items), Mental fatigue (seven items), Physical fatigue (six items), and Coping with fatigue (five items). DMFS is a promising tool to assist targeting of treatment to patients’ needs based on a detailed account of fatigue. However, to the authors’ knowledge, no validation of DMFS has been published to date other than the initial validation during development of the scale, which determined the factorial structure using principal component analysis and demonstrated evidence of internal consistency and convergent and divergent validity (Visser-Keizer et al., 2015). Furthermore, it has not yet been investigated how respondents interpret and respond to items of DMFS.

With reference to William James’ notion of the psychologist’s fallacy, i.e., the confusion of one’s own standpoint with that of others (Ashworth, 2009), Markus and Borsboom (2013) emphasized that test responders potentially interpret items differently than test developers intended. Consequently, test users risk drawing false inferences about the meaning of test scores, if the interpretative process involved in test responding goes unrecognized. In context of fatigue assessment, Mead et al. (2007) reported that some fatigue scales ask patients to rate the extent to which fatigue interferes with physical functioning, even though some stroke patients may not be able to disentangle the effect of fatigue from that of paresis. Furthermore, Tyson and Brown (2014) highlight the risk of conflating fatigue scores by including items that refer to the impact of fatigue on functioning and everyday activities, which are also directly limited by the neurological condition. The ambiguity of the concept of fatigue and the heterogeneity of the stroke population emphasize the need to evaluate validity of fatigue scales by elucidating the processes involved in item responding.

Since 1999, the Standards of Educational and Psychological Testing have described response processes as a source of validity evidence (American Educational Research Association. American Psychological Association, and the National Council on Measurement in Education, 2014). Hubley and Zumbo (2017) defined response processes as “the mechanisms that underlie what people do, think, or feel when interacting with, and responding to, the item or task and are responsible for generating observed test score variation” (Hubley and Zumbo, 2017, p. 2). Techniques of cognitive interviewing such as think-aloud and verbal probing procedures are well suited to elucidate response processes and provide evidence concerning the fit between observed response processes and the intended construct (Willis, 2005; Castillo-Díaz and Padilla, 2013; Padilla and Benítez, 2014; Launeanu, 2016).

In this validation study, we translated DMFS into Danish and evaluated how adults with stroke sequelae interpret and respond to items of DMFS. More specifically, objectives were to: (i) evaluate if participants respond to DMFS in ways expected; (ii) identify any difficulties responding to items; and (iii) identify themes elicited within subscales based on observed response processes. The study was part of a larger ongoing validation project on the Danish version of DMFS, and parallel research is currently being conducted to investigate psychometric properties of the present version using statistical procedures.

## Materials and Methods

This study comprised two stages. In the first stage, DMFS was translated into Danish using a back-and-forward procedure. In the second stage, construct validity of the Danish version of DMFS was evaluated using a Three-Step Test-Interview (TSTI) protocol (Hak and Jansen, 2008) to collect data and using Framework Analysis (Ritchie and Spencer, 1994) to transform and synthesize data. The study was conducted in concordance with the Declaration of Helsinki.

### Stage I: Translation of DMFS into Danish

DMFS was translated from English into Danish (Sechrest et al., 1972). Permission to translate DMFS was obtained from the first author of the original publication. Forward translations were completed independently by two researchers in ABI, who were native speakers of Danish and proficient in English (authors AN and TS). The two translations were reconciled into one consensus version by the forward translators. The forward translation was then translated back into English by a native speaker of English, who was experienced in back translations of patient-reported outcome measures. The back translation was then approved by the first author of the original publication.

### Stage II: Evaluation of Response Processes to the Danish Version of DMFS

#### Setting and Participants

Participants were recruited in an outpatient setting at BOMI Brain Injury Rehabilitation Center, Denmark. Inclusion criteria were as follows: (1) 18 years old; (2) fluent in Danish; (3) ABI; and (4) able to provide informed consent. Individuals were excluded if they had: (1) a progressive neurological disease such as brain tumor, (2) mild traumatic brain injury, or (3) overt cognitive difficulties interfering with the ability to respond on questionnaires (as evaluated by the interviewer). No criterion was exerted on time since injury, as all individuals considered for inclusion were in late stages of rehabilitation (10–34 months post-injury) due to the setting of the study.

Clinicians at BOMI were informed about the study and were instructed to refer adults with ABI, who were engaged in or had completed vocational rehabilitation. Adults referred to the study were screened for eligibility and provided with details about the study on telephone. If eligible, an interview was scheduled. Written informed consent was provided and background characteristics were obtained at the interview. Initial target sample size was 10 participants; however, recruitment stopped following nine interviews as data saturation was obtained. There is no standard sample size recommendations for cognitive interviewing, as the number of interviews needed depends on several factors (Terwee et al., 2018). COSMIN guidelines classifies at least seven participants as “very good” (Terwee et al., 2018), and Willis (2005) recommends between 5 and 15 interviews for a testing round before testing a revision of the questionnaire in another round.

#### Data Collection

Observation-based interviews were conducted using a TSTI protocol (Hak and Jansen, 2008). First, participants responded to DMFS while thinking aloud, i.e., reading items aloud and verbalizing thoughts as they respond to items. The interviewer kept records of response behavior (e.g., hesitation, contradictory use of the response anchor, skipping of items, gesture and interaction with the interviewer, etc.) and would only intervene to remind participants to think aloud. Second, upon finishing the questionnaire, the interviewer probed any unusual response behavior. Third, a semi-structured interview was conducted using an interview guide listing two to three optional follow-up probes for each item. A combination of general probes (e.g., “How did you arrive at that answer?”) and comprehension probes (e.g., “What does the term physical fitness mean to you?”) was used. The optional selection of probes during interviews was guided by saturation of data for subsequent coding.

Four interviews (P01–P04) were conducted by author FD, and five (P05–P09) were conducted by a trained master student in Psychology. Participants did not previously know any of the data collectors. Interviews were conducted face to face in a private room, either at the rehabilitation center or at the participant’s home. Interviews took about 75 min to complete, and all interviews were audio recorded.

#### Data Transcription and Transformation

Data were transcribed verbatim using Konch software.

Authors (FD and TA) independently coded data according to whether observed response processes were congruent with expected responses based on construct theory. First, the researchers established a common understanding of subscale constructs based on descriptions in the original publication of DMFS as summarized in Table 1 (Visser-Keizer et al., 2015). Notably, Physical fatigue was described to represent physical fitness in addition to fatigue experienced physically. However, we found it misleading regarding the title of the subscale, and thus participants were expected to refer to aspects of fatigue—and not solely aspects of physical fitness. Second, an index was designed based on the index used by Bunzli et al. (2018). The index comprised four categories:

(1) Congruent (i.e., observed response was related to the subscale construct);
(2) Incongruent (i.e., observed response was not related to the subscale construct);
(3) Ambiguous (i.e., observed response was both congruent and incongruent or insufficient to determine congruency);
(4) Confused (i.e., observed response was generated based on comprehension difficulties).

**Table 1:** Construct theory of subscales on the Dutch Multifactor Fatigue Scale.

Subscale name	Items	Construct theory, i.e., the scale addresses…
Impact of fatigue	11	“Frequency and severity of fatigue, general experience of fatigue, need to rest, and impact on daily life,” including “limitation of activities and greater emotional suffering due to fatigue”.
Signs and direct consequences of fatigue	9	“Symptoms that directly co-occur with fatigue, both emotionally and physically”.
Mental fatigue	7	“Fatigue experienced mentally after performing mentally demanding activities and hindering mental functioning,” including “precursors and consequences of mental fatigue”.
Physical fatigue	6	“Fatigue experienced physically and related to physical activities,” including “physical fitness and the precursors and consequences of physical fatigue”.
Coping with fatigue	5	“Coping with the limitations imposed by fatigue,” including “The ability of patients to signal fatigue and use this signal to adapt to fatigue”.

*Note. Descriptions of construct theory are quoted from Visser-Keizer et al. (2015)*.

Using the index above, both researchers coded all items for each transcript independently. Disagreements were settled through discussion after indexing of four transcripts and after another five. An agreement was reached in all instances.

#### Data Analysis

Distribution of coded response processes to items was illustrated and investigated using the “ggplot2” package in R (R Core Team., 2018). Items with less than 50% congruent responses were characterized as “problematic items” requiring more attention in analysis. This cutoff was based on a methodological evaluation by the authors to provide an overview of items with the most frequent problems.

Following coding of response processes, separate charts were constructed for each item. Columns contained index categories as headings, and rows contained individual participants. Next, author FD analyzed transcripts item by item following analytic steps described by Miller et al. (2014). First, summaries of each participant’s interpretation of items and any response difficulties were entered on the chart, including illustrative passages for possible quotation. Next, common themes across participants were synthesized for each item to identify “what the item captures,” both regarding congruent and incongruent response processes.

## Results

Twelve individuals were referred to screening from August to September 2019. One rejected to participate due to emotional distress, one did not reply to phone calls, and one was not eligible (brain injury due to tumor). Although multiple causes of ABI were eligible, no other eligible type of ABI than stroke was referred to the study. Thus, nine individuals with stroke participated in the study.

The sample comprised five females and four males (please see Table 2). Mean age was 55 years (SD = 6.3). All but one participant (P06) had a first-time stroke. Median time since injury was 26 months (range = 10–34). Mean scores on DMFS subscales were as follows: Impact of fatigue (11–55 score range) = 46.4, SD = 4.5; Signs and direct consequences of fatigue (9–45 score range) = 33.7, SD = 4.8; Mental fatigue (7–35 score range) = 30.6, SD = 3.2; Physical fatigue (6–30 score range) = 18.6, SD = 3.8; and Coping with fatigue (5–25 score range) = 15.2, SD = 5.4.

**Table 2:** Sample characteristics.

ID	Sex	Age (years)	Highest educational attainment	Type of stroke	Months since injury
01	F	59	Tertiary	Ischemia	33
02	F	45	Upper secondary (vocational)	Ischemia	34
03	M	46	Tertiary	Ischemia	10
04	F	54	Upper secondary (vocational)	Hemorrhage	29
05	M	62	Upper secondary (vocational)	Ischemia	26
06	M	61	Elementary	Ischemia	10
07	F	49	Tertiary	Ischemia and hemorrhage	32
08	M	58	Upper secondary (vocational)	Ischemia	11
09	F	56	Tertiary	Hemorrhage	26

*Note. Educational attainment was categorized using levels of the International Standard Classification of Education (ISCED) 2011 (UNESCO Institute for Statistics)*.

### Congruency of Response Processes With Subscale Constructs

**Figure 1:**
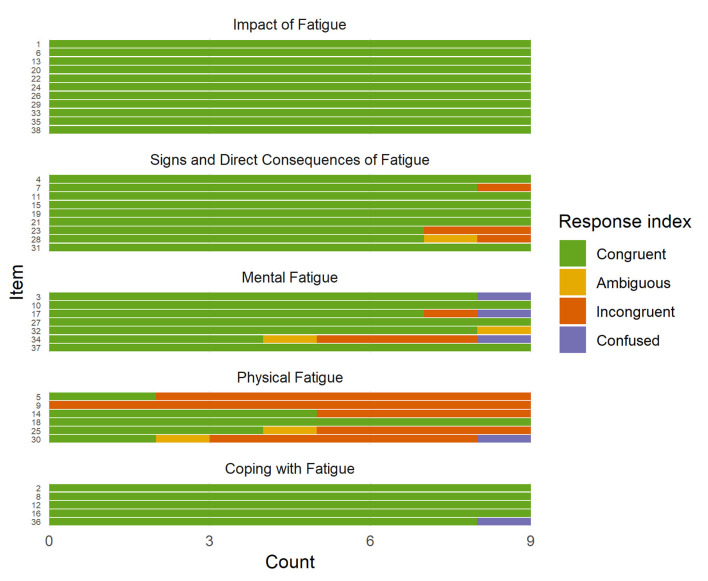
Distribution of indexed response processes of nine individuals with stroke to items of the Dutch Multifactor Fatigue Scale (DMFS). Note. The interpretative process in responding to items of the DMFS was indexed using the following categories: (i) Congruent (i.e., observed response was related to the subscale construct); (ii) Incongruent (i.e., observed response was not related to the subscale construct); (iii) Ambiguous (i.e., observed response was both congruent and incongruent or insufficient to determine congruency); and (iv) Confused (i.e., observed response was generated based on comprehension difficulties).

The distribution of indexed response processes is illustrated in Figure 1. All participants responded congruently to 25 out of 38 items. The Physical fatigue subscale had the highest proportion of incongruent response processes (54%). Five items were characterized as problematic items, i.e., less than 50% congruent responses (Table 3). Four of these were items of the Physical fatigue subscale and one was an item of the Mental fatigue subscale.

**Table 3:** Problematic items.

Item	Content	Subscale	Congruent responses
9	“I am in good physical condition”	Physical fatigue	0%
5	“I feel physically fit”	Physical fatigue	22%
30	“I have little energy”	Physical fatigue	22%
25	“My body aches when fatigued”	Physical fatigue	44%
34	“My complaints get worse when I am fatigued”	Mental fatigue	44%

*Note. Items with <50% congruent response processes were characterized as problematic items*.

#### Impact of Fatigue

All participants responded congruently to all items of the Impact of fatigue subscale. The intended construct of the subscale was defined as the frequency and severity of fatigue and the impact on daily life, including limitations in activities and emotional suffering due to fatigue (see Table 1). In responding to these items, participants referred to characteristics of fatigue, e.g., frequency, generation, and duration of fatigue, and impact of fatigue on everyday activities and well-being.

#### Signs and Direct Consequences of Fatigue

No items of the Signs and direct consequences of fatigue subscale indicated problems of congruency with the intended construct, i.e., “symptoms that directly co-occur with fatigue, both emotionally and physically.” In addition to items addressing manifestations of fatigue and co-occurring symptoms, response processes to three items (items 4, 11, and 31) referred to diurnal variations in fatigue and time needed to recover from fatigue.

#### Mental Fatigue

One item of the Mental fatigue subscale indicated potential problems of congruency with the intended construct, namely, item 34 “My complaints get worse when I am fatigued” (see Table 3). Three participants responded incongruently to this item as they described complaints unrelated to mental activities such as worsening of physical limitations or lack of initiative to do physical training when fatigued:

“If I am fatigued, then I need to get my walking stick because all of a sudden, I could risk that my legs disappear underneath me” (Participant 09).

Furthermore, one participant described complaints related to both physical and mental activities, and thus this response process was classified as “ambiguous.” Finally, one response process to item 34 was classified as “confused.” This participant had difficulties interpreting the term “complaints”:

“I do not get the question […] What are my complaints? What are complaints? Is it my physical problems, is it my financial—is it rent? What is it?” (Participant 08).

Consequently, analysis of response processes to item 34 revealed that four out of nine participants referred to complaints related to physical activities and that one participant got confused with the term “complaints.” Remaining items of the Mental fatigue subscale did not indicate issues of congruency with the intended construct.

#### Physical Fatigue

Of the six items of the Physical fatigue subscale, four were classified as problematic items, i.e., less than 50% congruent response processes, namely, items 9, 5, 25, and 30 (see Table 3). All participants responded incongruently to item 9, “I have a good physical condition.” In responding to this item, three participants referred to general health and chronic illness such as hypertension or susceptibility to the flu, and most participants referred to physical disabilities and paresis unrelated to fatigue, e.g.,

“If my stroke had involved a paralyzed arm or a paralyzed leg or something like that, then I would say that I have a bad physical condition” (Participant 08).

Most participants (7/9) responded incongruently to item 5, “I feel physically fit.” Similar to responses to item 9, three participants referred to paresis or other physical disability. Others referred to gain of weight, loss of muscular strength, cardiorespiratory fitness, or the ability to stand, walk, cycle, etc., without reference to fatigue.

About half of the participants’ responses (4/9) to item 25, “My body aches when fatigued,” were incongruent. Analysis of these response processes revealed that participants described being more attentive to chronic pain when resting, having common aches related to physical exertion, or having headaches, and one participant described that pain following physical exertion brought on her fatigue. One participant’s response was classified as “ambiguous.” This participant referred to both congruent and incongruent aspects, as he described having legs as heavy as lead and getting a headache when fatigued.

About half of the participants’ responses (5/9) to item 30, “I have little energy,” were classified as incongruent, another was classified as ambiguous, and another was classified as confused. Analysis of incongruent response processes to this item revealed that some participants described not having energy for as long as before injury to engage in daily activities. One participant described being more considerate of what she uses her energy for, and one described not being as inclined as before to engage in activities:

“Formerly, I was very sociable and outgoing, and now I rather just stay at home. […] I probably take more care of what I do, and what I not do” (Participant 02).

Thus, these participants did not refer to physical aspects of fatigue when responding to this item. One participant’s response was classified as “confused.” This participant found the item too vaguely defined and did not know how to interpret the term “little”:

“Compared to when I was healthy, I am probably at 50 percent. So that is not little, is it? What would you say?” (Participant 08).

In sum, these four items of the Physical fatigue subscale indicated problems, as most respondents did not refer to physical aspects of fatigue as intended. Regarding the remaining two items of the subscale, item 18 did not indicate any problems whereas item 14 demonstrated mixed results with four out of nine response processes being incongruent (see Figure 1).

#### Coping With Fatigue

No items of the Coping with fatigue subscale were classified as problematic items. In fact, all but one participant responded congruently to each item (see Figure 1). This one participant was confused about item 36, “I often let myself become overtired when circumstances demand it,” and had difficulties comprehending and responding to the item. In congruent response processes to items of this subscale, participants referred to planning of rests and daily activities and means of managing fatigue in everyday living.

### Difficulties Responding to Items

Seven items proved signs of response difficulties for some participants. First, item 24, “I do not need to have a rest to make it through the day,” was cognitively difficult to process for three participants due to the syntactic complexity. Two of them scored the item in contradiction with their intention without noticing, e.g., one verbalized: “Yes, I do [need to have a rest…]” and marked “Yes, I strongly agree.”

Second, three participants described that item 17, “A lot of stimulation, such as activity or noise, makes me fatigued,” was double-barreled. More specifically, they found that activity and noise affect fatigue in opposite ways, which posed difficulties to them on how to respond to the item, e.g.,

“I have not been to the cinema or the theater, because there is too much noise, too much light, and too many people […] Activities could be many things. If I go for a ride on the roller skates, that definitely does not make me fatigued. It makes me tired physically, but it does not make me tired mentally” (Participant 01).

Third, item 14, “After a good night’s sleep, I wake up rested,” presupposes that respondents experience a good night’s sleep, which was not the case for one participant. This participant responded “No, I strongly disagree” with reference to never having a good night’s sleep.

Fourth, three items included terms that were too vague to some participants. In response to item 3, “I can follow conversations without getting tired,” participants described that it depends on the kind of conversation and the number of people involved. Furthermore, in responding to item 7, “Emotional issues make me tired,” some participants distinguished the effect of positive emotions from negative emotions. When responding to item 15, “Other people notice that I am fatigued, before I do,” participants described that it depends on whom “other people” are, as those who know them well were more likely than strangers to notice them being fatigued.

Finally, regarding item 29, “Fatigue is my most serious problem,” the word “problem” was translated into a Danish term similar to “complaint,” which is commonly used by practitioners to denote patient-reported sequelae. However, some participants found this wording odd, as they did not see themselves as complaining, and were uncertain on how to interpret and respond to the item:

“I am not complaining, I mean, it [fatigue] is a part of me. That is just the way it is” (Participant 02).

Regarding layout of the questionnaire, one participant had difficulties keeping track of the items and skipped an item by accident several times, as the 38 items were too closely spaced on a single piece of paper (size A4).

### Contents of Congruent Response Processes

Table 4 summarizes main themes and subthemes within subscales identified during synthesis of congruent response processes to items of DMFS. Findings revealed overlaps in contents between some subscales, e.g., both Impact of fatigue and Signs and direct consequences of fatigue addressed duration of and recovery from fatigue. Furthermore, some subscales triggered a relatively broad range of topics and reasoning processes in responding. For instance, in responding to Coping with fatigue, respondents referred to different approaches to resting, to acceptance of limitations posed by fatigue, and to attending to own needs. Furthermore, respondents also described conflicting inclinations to finish activities in hand, to keep appointments and avoid disappointing others, and to attend family gatherings and other activities, which were considered important but were expected to trigger fatigue.

**Table 4:** Themes identified within subscales in synthesis of congruent response processes to items of the Danish version of the Dutch Multifactor Fatigue Scale.

Subscale	Main themes	Subthemes	Example
Impact of fatigue	Nature of fatigue	Frequency	Daily experience
		Severity	Disabling and debilitating effects on everyday living
		Generation	Sudden and unpredictable
		Duration	Recovery time, potentiality for relief
	Impact on activities	Limited amount	Vocational, leisure, and rehabilitation
		Way of engaging	Need for planning, lack of spontaneity
		Need for rest	Regular rests or as needed
	Impact on well-being	Acceptance	Coming to terms with fatigue
Signs and direct	Co-occurring symptoms	Headache
consequences of fatigue		Excessive thoughts	Worries, rumination, and unrest
		Irritability	Becoming frustrated, worried, and upset easily
	Nature of fatigue	Diurnal patterns	Accumulates during the day
		Duration	Recovery time, potentiality for relief
		Visibility	Close relatives may notice fatigue
Mental fatigue	Manifestation of fatigue	Attention problems	Focusing attention, concentrating, distractibility, inattentiveness, losing the thread of a conversation
		Problem solving	Making simple mistakes and spending more time getting things done
	Triggers	Mental exertion	Planning and extended concentration
		Sense impressions	Noise, light, and bustle
Physical fatigue	Manifestation of fatigue	Weakness	Increased perceived exertion in simple physical activities such as walking
		Pain	
	Physical activity	Exacerbate fatigue	Physical work or exercise; Experienced physically as pain or weakness
		Relieve fatigue	Getting fresh air and feeling tired physically as opposed to mentally; Improved mood from working out
Coping with fatigue	Coping strategies	Attending to own needs	Showing respect for and adapting to fatigue in everyday living; Backing out of activities if needed
		Settling for less	Knowing one’s limits and adjusting ambitions and liabilities
		Scheduling rests	Planning of rest with due consideration of scheduled activities
		Resting as needed	Attending to sensations of fatigue and having rests as needed
	Trigger mechanisms	Exceeding the limit	Inclined to finish activities in hand; Having to complete daily chores; Valuing important activities above the costs of overexertion; Disinclined to cancel appointments and disappoint others

## Discussion

This study investigated how adults with stroke sequelae interpret and respond to items on the Danish version of DMFS. While most items performed as intended, five items demonstrated potential problems regarding construct validity, including four items of the Physical fatigue subscale. In addition, seven items posed various responding difficulties to some respondents. Finally, common themes in congruent response processes were identified within subscales.

Four out of six items of the Physical Fatigue subscale demonstrated problems with regard to construct theory. Problems were that items not only addressed a concept of physical fatigue but also related concepts of health issues, physical disability, and general physical condition. Consequently, the scale score is at risk of conflation with extraneous factors, which may lead to false inferences about what the score represents. When using the DMFS for clinical or scientific purposes, careful consideration of the construct of this subscale is needed. Furthermore, if the DMFS is to be used with the intention of assessing a unitary concept of physical fatigue, the present results would suggest a substantial revision of subscale items. However, it may be of clinical relevance to assess a broader concept of physical functioning, including any disabilities, to inform decisions about therapeutic targets and feasibility of intervention strategies, e.g., capability of initiating physical training. In this case, however, construct theory needs refinement and the title of the subscale may need revision, as it currently indicates a measure of physical fatigue in the strict sense.

The remaining subscales of the DMFS performed well in terms of response processes, as participants interpreted and responded to items in ways expected. Findings indicate that the DMFS addresses a wide range of aspects in relation to fatigue, including the nature of fatigue, different types of precursors, manifestations, and co-occurring symptoms, and the impact of fatigue on everyday living and well-being. In general, the DMFS was comprehensible and items were relevant to participants, and some participants faced questions about their fatigue, which they had not considered before.

A distinct feature of the DMFS, compared to most other fatigue scales used in stroke populations, is the separate measure of coping with fatigue (Skogestad et al., 2019) and its promising prospects of targeting and evaluating treatment (Visser-Keizer et al., 2015). Although being congruent with the intended construct, response processes to items of the Coping with fatigue subscale indicated possible challenges regarding causal theory of the scale score. For instance, some participants described deliberate planning of rest with due consideration of scheduled activities and expected exertion, while others described preferring to rest as needed throughout the day, as this strategy was perceived more beneficial to them. Interestingly, the latter tended to disagree in responding to item 2, “I consciously plan when I will rest,” resulting in a higher scale score regardless of whether this strategy is helpful or not for the particular subject. Consequently, careful consideration is needed when interpreting the scale score.

Furthermore, the scales of the DMFS ought to be interpreted together, and Coping with fatigue may have limited use if evaluated in and of itself, as coping efforts may respond to contextual changes (Lazarus and Folkman, 1984). For instance, consider an individual experiencing improved vitality and less impact of fatigue following rehabilitation. Due to alleviation of fatigue, this individual may not need to adapt everyday life to fatigue to the same extent as before rehabilitation and thus would be likely to get a higher score on Coping with fatigue (indicating lesser ability to cope with limitations imposed by fatigue). If the diminished need to cope with fatigue goes unrecognized, the score could be misleading. Interpretation of Coping with fatigue is further complicated as some participants described occasions of deliberately prioritizing to do things that they know will make them tired. For instance, some described valuing the benefit of participating in activities considered being important to them, e.g., attending family gatherings, above the cost of associated overexertion and concomitant symptoms of fatigue.

Consequently, Coping with fatigue may not represent the *ability* to cope with fatigue *per se*, but rather the extent to which respondents perceive themselves as taking fatigue into account and adapting everyday life to limitations imposed by fatigue. In broader perspective, standardized assessment of coping is a challenging task, as different strategies may be effective to different individuals in different circumstances, and widely used measures of coping often comprise inventories of multiple coping strategies (Greenaway et al., 2015). Furthermore, personal costs associated with adjusting everyday life to fatigue may exceed perceived benefits in some instances.

In addition to considerations regarding construct theory, results indicated a few problems regarding comprehension and responding to items, which require attention prior to release of the Danish version or any new round of testing of a revised version. However, it is not clear whether issues identified in this study are pertinent to the Danish translation of the DMFS constituting translation errors, or if they reflect general problems of the original DMFS. As response processes have not been evaluated for other versions of the DMFS, any general problems are unknown, and issues identified in this study are potentially prevalent in other versions as well, especially as the DMFS was translated using a rigorous procedure. Cognitive interviewing of both the Danish and the original version will elucidate whether problems are related to translation errors, to the source questionnaire, or to any issues of cultural portability (Schoua-Glusberg and Villar, 2014).

Altogether, results reflect the ambiguity of the concept of fatigue and emphasize the need for researchers and clinicians to inspect the performance of items in any self-report measure of fatigue. As fatigue is a vague concept, the terms used to address fatigue may easily be interpreted in different ways than intended. Involving patient perspectives in test development and evaluation, e.g., by use of cognitive interviewing techniques, may shed light on interpretative processes involved in test responding and aid refinement of self-report measures of fatigue and interpretation of test scores.

From a clinical perspective, one purpose of assessment is to inform treatment planning and aid clinical reasoning behind the continuous decision-making on targets and means to achieve rehabilitation goals. In this regard, the DMFS gives a detailed and multidimensional account of fatigue, including coping with fatigue, which offers information about different aspects of fatigue and ways of managing fatigue. However, the utility of this instrument for targeting treatment has not been established, and it may be promising for future research to investigate how the DMFS may guide treatment. Furthermore, as the DMFS is a rather comprehensive questionnaire, the prospect of a short version, while retaining the multidimensional structure, may be beneficial to save resources and to avoid unnecessary strain on easily fatigued individuals. Future research on item information and redundancy, e.g., by the use of confirmatory factor analysis and item response theory, is required in this respect. As some individuals with stroke may have visual deficits or visuoperceptual difficulties, questionnaire layout may also be revised to mitigate any response difficulties or non-responses to items on this basis.

### Methodological Considerations

A few methodological considerations require attention. First, sample size target was determined *a priori* based on identified available guidelines (see Willis, 2005; Terwee et al., 2018). After completing nine interviews, a number of concerns were raised regarding the DMFS, and we evaluated saturation and found it satisfactory. However, recruiting more participants may have elucidated more subtle problems. Based on the present results, further cognitive interviewing is recommended for any future revisions of the DMFS as for other versions of the questionnaire.

Second, the present study included individuals with stroke only. However, target population of the DMFS is mixed ABI, and items may perform differently to individuals with non-vascular types of ABI. The nature of fatigue may differ in some ways across various health conditions (Eilertsen et al., 2015; Whitehead et al., 2016), and specific characteristics of other types of ABI such as demographics or sequelae may affect the way respondents interact with items. Consequently, results may not be generalizable to all subgroups of the target population of the DMFS. Furthermore, sample characteristics such as stroke severity and stroke sequelae, e.g., cognitive deficits and physical limitations, were not systematically assessed for comparison with other research studies. In this respect, the present validation in stroke is an important first step, and future research is needed to investigate evidence of any subgroup variation in the ABI population.

## Conclusion

This study provides validity evidence based on response processes, identifies potential problems in item performance, and elucidates concepts captured by each subscale for the Danish version of the DMFS as a multidimensional measure of self-reported fatigue post-stroke. While most items performed well in addressing aspects of fatigue, results indicate conflation of the Physical fatigue subscale with extraneous factors such as physical disability due to stroke sequelae and general health conditions. Findings guide any amendments that may be considered to mitigate problems identified in cognitive interviewing and to improve the performance of the instrument. While issues may constitute translation errors, the possibility of issues in other versions of the DMFS cannot be ruled out. In addition to further validation, prospects for future research include investigation of the utility of a short version, more detailed specification of construct theory, and evaluation of the utility of the DMFS for guiding and targeting rehabilitation of fatigue and associated factors.

To the authors’ knowledge, this is the first study to investigate the interpretative processes involved in responding to a self-report measure of fatigue in brain injury populations. While this study contributes with evidence to support the validity of the DMFS and guides modifications to mitigate potential issues of item performance, these findings also emphasize the utility of this methodological approach in scale validation and the need for more thorough evaluation of existing measures of fatigue.

## Data Availability Statement

The raw data supporting the conclusions of this article will be made available by the authors, without undue reservation.

## Ethics Statement

Ethical review and approval was not required for the study on human participants in accordance with the local legislation and institutional requirements. The patients/participants provided their written informed consent to participate in this study.

## Author Contributions

AN and TS translated the questionnaire. TA and FD prepared the interview guide and other procedures for data collection. TS and FD prepared procedures for recruitment of participants. FD recruited participants and collected data. FD recruited participants and collected data; performed descriptive statistics, thematic analyses, and prepared the first draft of the manuscript; organized data, and FD and TA analyzed data. All authors reviewed methods and results critically and contributed to the interpretation and implications of findings. All authors revised the draft of the manuscript critically. All authors contributed to the article and approved the submitted version.

## Conflict of Interest

The authors declare that the research was conducted in the absence of any commercial or financial relationships that could be construed as a potential conflict of interest.
